# Preadolescent Students’ Engagement With an mHealth Intervention Fostering Social Comparison for Health Behavior Change: Crossover Experimental Study

**DOI:** 10.2196/21202

**Published:** 2021-07-29

**Authors:** Raoul Ceasar Yannic Nuijten, Pieter Van Gorp, Tom Borghouts, Pascale Le Blanc, Pauline Van den Berg, Astrid Kemperman, Ehsan Hadian, Monique Simons

**Affiliations:** 1 Department of Industrial Engineering Eindhoven University of Technology Eindhoven Netherlands; 2 Department of the Built Environment Eindhoven University of Technology Eindhoven Netherlands; 3 Department of Social Sciences Wageningen University and Research Wageningen Netherlands

**Keywords:** mHealth, health promotion, social comparison, competitiveness, collaboration, gamification, preadolescents, high school students

## Abstract

**Background:**

Contemporary mobile health (mHealth) interventions use various behavior change techniques to promote healthier lifestyles. Social comparison is one of the techniques that is consensually agreed to be effective in engaging the general population in mHealth interventions. However, it is unclear how this strategy can be best used to engage preadolescents. Nevertheless, this strategy has great potential for this target audience, as they are particularly developing their social skills.

**Objective:**

This study aims to evaluate how social comparison drives preadolescents’ engagement with an mHealth app.

**Methods:**

We designed a 12-week crossover experiment in which we studied 3 approaches to implementing behavior change via social comparison. This study was hosted in a school environment to leverage naturally existing social structures among preadolescents. During the experiment, students and teachers used an mHealth tool that awarded points for performing healthy activities. Participants could read their aggregated scores on a leaderboard and compare their performance with others. In particular, these leaderboards were tweaked to implement 3 approaches of the social comparison technique. The first approach focused on intragroup comparison (ie, students and teachers competing against each other to obtain the most points), whereas the other two approaches focused on intergroup comparison (ie, classes of students and their mentoring teachers collaborating to compete against other classes). Additionally, in the third approach, the performance of teachers was highlighted to further increase students’ engagement through teachers’ natural exemplary function. To obtain our results, we used linear modeling techniques to analyze the dropout rates and engagement levels for the different approaches. In such analyses, we also considered individual participant traits.

**Results:**

Our sample included 313 participants—290 students (92.7%) and 23 teachers (7.3%). It was found that student engagement levels dropped over time and declined during holidays. However, students seemed to monitor the intergroup competitions more closely than the intragroup competitions, as they, on average, checked the mHealth app more often when they were engaged in team-based comparisons. Students, on average, performed the most unique activities when they were engaged in the second intergroup setting, perhaps because their teachers were most active in this setting. Moreover, teachers seemed to play an important role in engaging their students, as their relationship with their students influenced the engagement of the students.

**Conclusions:**

When using social comparison to engage preadolescents with an mHealth tool, an intergroup setting, rather than an intragroup competition, motivated them to engage with the app but did not necessarily motivate them to perform more activities. It seems that the number of unique activities that preadolescents perform depends on the activeness of a role model. Moreover, this effect is amplified by preadolescents’ perceptions of closeness to that role model.

## Introduction

### Research Case

Many people with chronic diseases and other health-related problems may benefit greatly from increased physical activity and improved dietary intake [1]. To support individuals adopting these healthier routines, behavior change interventions, including various behavior change techniques to encourage certain targeted behaviors, may be used [2]. The aim of this study is to strengthen the empirical evidence on the impact of one specific technique (ie, social comparison) for engaging a specific target group—in our case, preadolescents (ie, 10- to 13-year-olds).

We have chosen to target preadolescents because application of interventions at this stage in life will likely also impact health at a later stage, particularly because newly adopted lifestyle behaviors will track into adulthood [3]. By the time of preadolescence, the human brain is still developing, particularly strong in the areas of social skills and peer relationships [4]. Therefore, when designing an intervention for preadolescents, taking into account the social dynamics within that target group is of vital importance for intervention effectiveness.

At the same time, social dynamics have been used to foster health behavior changes. Social comparison is one of the behavior change techniques that is consensually agreed to be effective for the general population [2]. This intervention strategy stems from the idea that, by nature, people tend to self-evaluate by comparing themselves with others [5]. Comparing oneself is a universal process that all of us engage in regularly, although some more than others [6]. Similarly, competitive processes, as manifestations of social comparison [7], are prevalent in our societies [8]. As these phenomena are common in everyday life, it seems beneficial to evaluate whether these processes can be applied to promote health behavior change.

In mobile health (mHealth) interventions, the adoption of leaderboards offers an opportunity to implement social comparison and competitiveness as intervention strategies [9]. Leaderboards are a form of gamification, a set of motivational techniques that use game mechanics outside game contexts, to foster participation, engagement, and loyalty [10,11]. Leaderboards may be used to increase participant engagement and are widely adopted as one of the most popular gamification techniques [12]. In this study, we investigate how to design such leaderboards for the optimal engagement of preadolescents.

The degree to which individuals are engaged in a particular social comparative setting is determined by different situational and individual factors [8]. A key decision when designing such a setting is to determine whether comparative and competitive processes occur either intra- or intergroup [13]. In an intragroup setting, individuals compete against each other, whereas in an intergroup setting, groups of individuals collaborate to compete against other groups.

In this study, we evaluate the implementation of social comparison (ie, either fostering intra- or intergroup comparisons) that is most effective in promoting healthy routines in preadolescents. As social dynamics among preadolescents are likely articulated in their school environment and because the World Health Organization has put forward the key objective of “[generating] scientific evidence on effective Health Promoting School (HPS) interventions” [4], we have implemented our intervention at a high school. In this environment, educational levels and classes are the main social structures. Moreover, teachers have an exemplary function within a school environment and potentially serve as positive role models for students [14]. In our experiment, we analyzed the role of social dynamics between students and teachers in three study arms.

We hypothesized that an intergroup approach, which combines collaborative and competitive aspects, would be engaging for students and would encourage them to adopt new healthy routines. We based our hypothesis on the observation that an intergroup approach can potentially trigger processes of self-enhancement in children and processes of enhancement of others, whereas intragroup competition is likely to solely promote self-enhancement, potentially even at the expense of others [15,16]. This claim is supported by a recent review of competitive versus cooperative aspects in social exergames: cooperative play can “increase motivation, promote continued play, and increase prosocial behaviors,” whereas competitive play mainly yields short-term, physiological arousal [17]. To empirically evaluate whether the potential spillover effects of intergroup competitions do indeed positively influence students’ engagement levels in a health promotion campaign, we designed study arms that ranged from more intragroup to more intergroup focused. Before explaining these treatments in detail in the *Methods* section, the factors influencing the social comparative behavior are evaluated in depth.

### Theoretical Background

By nature, people tend to self-evaluate by comparing themselves with others [5]. This study evaluates how this natural tendency may be leveraged to sustain the engagement of preadolescent students in a health promotion campaign. Comparing ourselves with others may occur in different directions; we may compare ourselves, based on a specific aspect, with others who are worse (ie, downward comparison) or better (ie, upward comparison). Both downward and upward comparisons may affect one’s self-concept [7] and can foster competitive behavior [8]. Downward comparison will often enhance the self-concept of the comparator [18]. However, as downward comparison reveals that ones' status could decline if others catch up, the feeling of being threatened and discouragement might also be evoked [18]. Comparing ourselves with a superior other might cause negative feelings too, as the other performs better on certain attributes [19]. At the same time, upward comparison can lead to the assimilation of the characteristics of the superior other and provide hope and inspiration, especially if the superior is a role model (eg, a student’s teacher) [20].

The social comparison model of competition describes the factors that influence competitive behavior [8]. The model proposes that competitive processes are influenced by situational factors. For example, it was found that the number of competitors is best kept as low as possible: the lower the number of competitors, the more intense the competition (ie, the N effect [21,22]). Furthermore, it was found that incentives, such as tangible rewards, increase people’s engagement in a competitive setting [8,23,24].

In addition, the social comparison model of competition proposes that competitive processes are influenced by individual factors. For example, it was found that, for a competition to be engaging, participants have to perceive the dimension of comparison as relevant to the self [25]. This effect is particularly amplified if competitors perceive their relationships as *close* [26,27]. Furthermore, it was found that when competitors, either as a group or as individuals, share similar characteristics (eg, race or education), the competition intensifies [7,28]. Similarly, personality traits are known to increase competitiveness. In particular, social comparison orientation [6], competitive dispositions [29], and individuals’ orientation toward performance goals [30,31] seem to influence competitiveness. Moreover, the personality trait openness to experience (ie, as defined by the Big Five personality framework [32]) is a potential trait that can influence competitiveness: people who score lower on this trait and are therefore less independent and creative may be more competitive [33].

Finally, it has been previously demonstrated that especially intergroup competitions can enhance engagement in an activity [13] because it includes a mix of collaborative and competitive aspects. It was found that an intergroup competition can potentially trigger processes of self-enhancement in children and processes of enhancement of others, whereas intragroup competitions are likely to solely promote self-enhancement, potentially even at the expense of others [15,16]. These principles were not yet tested with preadolescents in schools.

This study aims to further such theoretical insights via a study design that contrasts inter- and intragroup competition in schools and that tests whether teachers as role models can increase engagement. Potential moderation of situational factors and personality traits is accounted for.

## Methods

### Recruitment

Participants were recruited among first-year, prevocational (ie, VMBO [Voorbereidend Middelbaar BeroepsOnderwijs]) students (ie, 11- to 13-year-olds) at a high school in the Netherlands in April 2019. The study was advertised as a health promotion campaign and conducted only after obtaining explicit written consent of the participants (ie, the students) and their parents or guardians. Explicit consent of the students was collected upon registration for the campaign. Explicit consent from their parents or guardians was collected via consent letters that they signed and returned to the school’s administration. More operational procedures were also approved by the ethical committee of Eindhoven University (Archie experiment ID 920). The ethical review committee concluded that the potential benefits of this study outweighed its potential risks. However, besides the potential positive impact of social comparison on health behavior, it was acknowledged that the target group may have also experienced the negative effects of social comparison (eg, feeling threatened or discouraged [18]). Meanwhile, it was found that the school environment provided a sufficiently safe setting to host this experiment, especially because teachers were advised to check in weekly with their students on the impact of the campaign. As such, the more vulnerable students could have been identified and corrective actions could have been taken.

### Intervention

To test our hypotheses, we have used the mHealth tool GameBus that is manufactured and maintained by Eindhoven University of Technology. GameBus was especially designed for health promotion and provides a highly configurable gamification engine that is used to sustain participants’ engagement. According to the classification of gamification elements by Hamari et al [12], GameBus implements the gamification mechanisms *challenges*, *points*, *goals*, *progress*, *leaderboards*, and *rewards* and configures these mechanisms to test scientific hypotheses. The tool supports hosting multiple experimental designs on a single platform, ensuring that user experience remains similar across these different designs. At the same time, the platform enables researchers to gather rich data in a manner compliant with European privacy legislations. For this study, a dedicated Privacy Impact Assessment was approved by the Data Protection Officers of Eindhoven University of Technology and the high school.

GameBus includes a mobile app (ie, available via the web as well as on Android and iOS) for tracking healthy activities. The platform is designed to allow rewarding any healthy activity with points, from social, to mental, to physical activities. For this study, the rewarded activities have been defined in consultation with the school's management and a student council. Several cocreation sessions were held with the aim of defining activities that students were capable of performing, that they would enjoy doing, and that would benefit their health. The mHealth tool would then reward students for performing these activities, based on a selfie as proof of conducting the activity.

Users could compare their performance on a leaderboard that summed up the points per user. In addition, for the intergroup approaches, additional leaderboards showed per team (ie, per class) the average number of points across team members. The app also provided a newsfeed, which showed an entry for each team member that scored points. Such entries could be liked or commented upon in a manner similar to mainstream social media platforms such as Facebook and Instagram.

The overall goal of the intervention from the students’ perspective was to obtain as many points as possible by adopting healthier routines. In particular, it was set by the school's management to focus on (1) increasing physical activity; (2) promoting healthy nutrition; (3) fostering sustainable relationships: friends, love, and intimacy; and (4) emphasizing the (potential) impact of stress, drugs, alcohol, and gaming. From these focal areas, a list of prescribed activities was compiled in consultation with the school’s management and a student council. The aim was to define activities that students were capable of performing, that they would enjoy doing, and that would benefit their health (eg, “Wrestle arms with someone of at least 40+” or “Peel an (unbroken) apple peel of at least 20 centimeters”), resulting in a list of 51 unique activities. These activities were distributed over the course of the campaign and renewed every *wave* (ie, every 2 weeks; a complete overview of prescribed activities per wave is provided in Multimedia Appendix 1). The entire campaign lasted 12 weeks (ie, 6 waves). Eventually, the first wave consisted of 12 unique activities, and in the other five waves, nine activities were prescribed. Each wave included a mix of the focal areas. Some activities were duplicated over multiple waves (a detailed overview is provided in Multimedia Appendix 1).

### Study Design

#### Treatment Allocation

From a scientific perspective, the intervention included three different social comparative settings as treatments, to test whether an intergroup—rather than an intragroup—competition would be more effective in promoting healthy routines in preadolescent students. A crossover study design was adopted to ensure that all the participants were exposed twice to every treatment. We adopted a randomized block approach to randomly distribute the treatments to the participants. By order of the school's management, the three clusters that this study design required were defined based on educational level (Dutch prevocational education distinguishes three such levels). Participants received the treatments in 2-week periods (ie, in so-called waves). The entire campaign lasted for 12 weeks (ie, 6 waves); therefore, each participant received every treatment twice.

Our treatments effectively simulated three different implementations of the social comparison technique. One approach represented an intragroup competition, whereas the other two approaches represented intergroup competitions. In one of these intergroup competitions, an additional comparative element was introduced (ie, by explicitly highlighting the performance of teachers), as this manipulation was expected to increase students’ engagement levels even further, because teachers potentially serve as role models for students and may, therefore, foster hope and inspiration in students during the competition, particularly if they perform (somewhat) better than their students [20]. Note that, although the treatments were different in nature, the overall objective was always the same from the participants’ perspective—to collect as many points as possible by performing the healthy activities prescribed in each specific wave. The player, or team, with the greatest number of points at the end of a wave (ie, the absolute winner) was awarded a small gift (ie, either a medal or a stress ball). The following paragraphs describe the different treatments in detail. Table 1 displays how the treatments were distributed across the participants. The rows distinguish between the three treatment groups. In the columns, it can be read what treatment each treatment group is assigned in a given wave.

**Table 1:** Distribution of treatments over participants and waves.

Educational level	Wave 1	Wave 2	Wave 3	Wave 4	Wave 5	Wave 6
Educational level A	SCS2^a^	SCS1^b^	SCS3^c^	SCS2	SCS1	SCS3
Educational level B	SCS3	SCS2	SCS1	SCS3	SCS2	SCS1
Educational level C	SCS1	SCS3	SCS2	SCS1	SCS3	SCS2

^a^SCS2: second social comparative setting.

^b^SCS1: first social comparative setting.

^c^SCS3: third social comparative setting.

#### First Social Comparative Setting: Intragroup Competition

**Figure 1:**
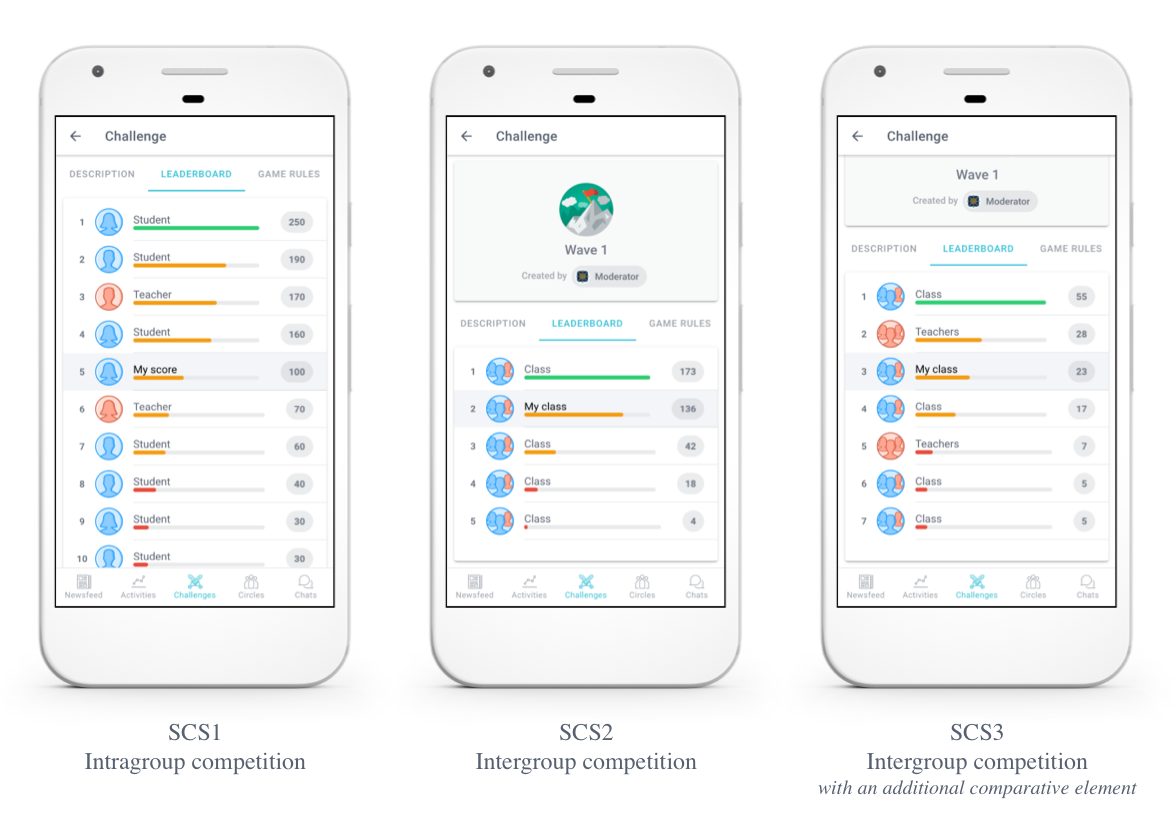
Leaderboard view of different treatments (students’ and class’ names removed for the sake of confidentiality). SCS1: first social comparative setting; SCS2: second social comparative setting; SCS3: third social comparative setting.

In the first social comparative setting (SCS1), students and teachers of a treatment group (ie, educational level) competed individually. In this intragroup competition, players could read each other’s performance from a leaderboard but could not see the actual activities, other than their own, that were performed to accumulate this score in the newsfeed of the mHealth tool (Figure 1). Only the absolute winner was awarded a small gift for this competition type.

#### Second Social Comparative Setting: Intergroup Competition

In the second social comparative setting (SCS2), students of a particular class and their mentoring teachers joined forces to compete against other classes within their treatment group (Figure 1). In this intergroup competition, players could read their class’ performance (ie, the average number of points collected by the members of their class) on the leaderboard. In addition, they could read their own contribution to the score of their class relative to the contribution of their class members and mentors, but they could not read the individual contributions of students and mentors of other classes. Similarly, players could see the actual activities that others performed to accumulate their score if, and only if, that player was within their own class. At the end of the wave, the entire winning team was awarded a small gift.

#### Third Social Comparative Setting: Intergroup Competition With an Additional Dimension of Comparison

The third social comparative setting (SCS3) closely resembled the second treatment: this setting also featured an intergroup competition in which the entire winning team was awarded a small gift at the end of a wave. In SCS2 however, students could not transparently compare their performance with their teachers (other than their own mentoring teachers) because a teacher’s score was concealed in the average score of the class they mentor and can, therefore, not be read by students from other classes. However, Lockwood and Kunda [20] argue that students can draw inspiration from the act of comparing themselves with their teachers, especially if their teachers (slightly) outperform them (ie, triggering upward comparison) [20]. Therefore, to allow students to compare themselves with their teachers, SCS3 included two extra teams that were composed of teachers only (Figure 1). These two teams included teachers who mentor students from the two other treatment groups (ie, the two other educational levels). Therefore, in SCS3, students would collaborate with their class members and mentoring teachers to compete against other groups of students and teachers.

### Study Procedures

At the start of the campaign, a kick-off day was scheduled. Before this day, teachers were introduced to the mHealth app and study context. Subsequently, the teachers instructed their students on using the mobile app. Throughout the kick-off day, a dedicated support team was present to assist students with installing the mHealth app. In addition, several workshops were organized by the Public Health Services to introduce students to topics such as healthy nutrition, the dangers of smoking, alcohol abuse, and the use of drugs.

To keep the campaign under the attention of students for the entire 12-week period, teachers were instructed to discuss their students’ progress in class once a week. It was particularly suggested to review in plenary class sessions the leaderboard and discuss the activities the students had performed over the last week. Unfortunately, this review could not take place in the third and fourth weeks (ie, during the second wave) of the campaign owing to the spring break.

### Measurements

In mHealth, engagement is most commonly captured via passive measures of app use [34]. Using the GameBus platform, engagement of participants was repeatedly measured as (1) the number of days a participant had visited the app, and (2) the number of unique activities a respondent performed. These variables complement each other because the former may be limited to passive engagement, whereas the latter requires active participation (ie, performing healthy activities). Note that our second outcome variable measures the number of unique activities a respondent performed rather than the total number of activities that were performed. The main reason is that we aim to encourage preadolescents to adopt a multitude of healthy routines, not just repeat a single routine (ie, quality over quantity). However, our results and conclusions did not differ when analyzing the total number of activities that respondents performed instead.

In addition, participants (ie, students only) filled out a posttest survey (disclosed in Multimedia Appendix 2) in which their propensity toward the individual factors proposed by the social comparison model of competition [8] was assessed. Specifically, students’ perception of closeness to their teachers, students’ perception of closeness to their peers, students’ perception of similarity to their teachers, students’ perception of similarity to their peers, students’ perception of relevance of the prescribed activities, and students’ personality were assessed. To assess their personality, respondents completed a personality test in accordance with the Big Five personality traits [32]. The posttest survey was completed by 112 students.

### Statistical Analysis

The first set of statistical analyses focused on the evaluation of dropouts. A respondent is labeled as a (provisional) dropout if the respondent has not visited the app in a given wave and is assumed to have lost interest (ie, dropped out) in the wave before. Several multiple regression models were fit to determine whether the number of dropouts changed over time and were different for each treatment. Subsequent analyses were performed on a subset of respondents who have participated in the study since the start of the first wave.

The second set of analyses focused on the evaluation of the engagement levels of both students and teachers. To evaluate treatment differences, further analyses were performed on respondents who actually had a chance to get exposed to the treatment. Therefore, from the entire data set, a subset was derived by preserving the combination of a particular respondent and wave only if the respondent had ever checked the app in that wave. Subsequently, several hierarchical linear models were estimated for the two outcome variables (ie, the number of days a participant had visited the app and the number of unique activities a respondent had performed) using time (ie, wave number), holiday, and treatment as predictors. We tested whether significant second-order interaction effects existed among these variables. In all models, we allowed random intercepts for both individuals and the classes they were in. The final model was selected based on Akaike information criterion [35]. Subsequently, in the final models, posthoc tests using Tukey adjustments were performed on the treatment variable. The same procedure was repeated to separately fit a model for students and teachers.

Finally, the third set of analyses focused on evaluating the impact of individual factors on engagement levels. Data on individual factors were derived from a posttest survey that was filled out by students only and not by teachers. Survey observations were linked via email addresses to GameBus user accounts to match individual factors with engagement levels. Although 112 students completed the survey, 10 responses could not be traced back to actual users of the mHealth platform. Furthermore, to evaluate the impact of individual factors, analyses were again performed on respondents who had a chance to get exposed to the treatment. Therefore, from the entire data set, a subset was derived, preserving the particular respondents that had checked the app over the course of the entire campaign at least twice, leaving 67 respondents in the data set for further analyses. Note that, in contrast to the second set of analyses, data are now aggregated over the course of the entire campaign and not per wave. Subsequently, several multiple regression models were fitted for the two outcome variables using the 10 individual factors as predictors. On the basis of Akaike information criterion, a backward selection procedure was used to select the final model [35].

## Results

### User Statistics

**Figure 2:**
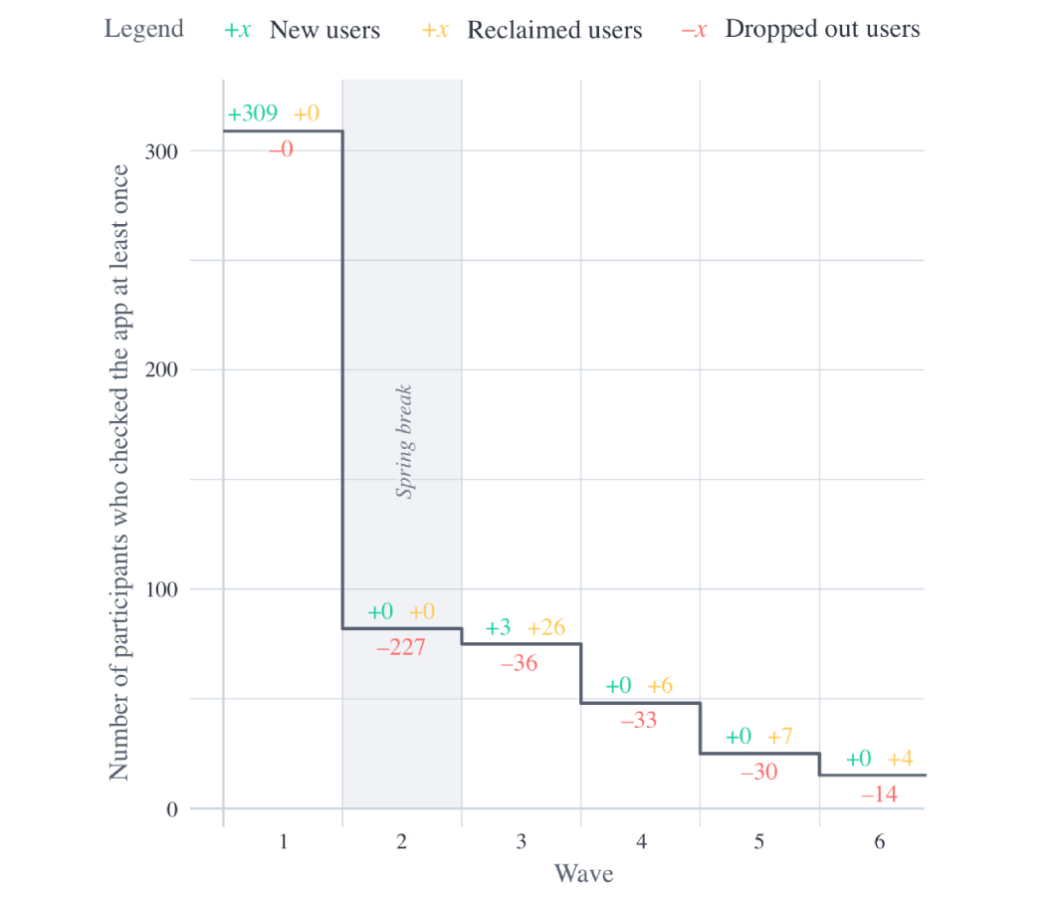
Number of participants who checked the app (at least once) per wave.

In total, 313 unique participants, including 290 students (92.7%) and 23 teachers (7.3%), participated in the study. Educational level A included 61 students and 6 teachers, educational level B included 110 students and 9 teachers, and educational level C included 119 students and 8 teachers. Figure 2 displays the degradation of the number of participants who checked the mobile app during a given wave. The number of participants who joined the campaign for the first time in a given wave is displayed in green. The number of participants that dropped out in a given wave is shown in red. The number of participants who checked the mobile app in a given wave, although they dropped out in an earlier wave (ie, reclaimed users), is displayed in yellow. It was found that students tended to drop out, especially at the beginning of the campaign (ie, the wave number was significant at *P*=.003) and during holidays (*P*=.04). No significant differences in dropout rates within treatments were detected. Therefore, it is assumed that dropouts are spread equally over treatments.

### Evaluation Outcomes

A total of 3 respondents only joined in the third wave and, therefore, were excluded from further statistical analysis, leaving a total of 99% (288/290) of students and 95.5% (22/23) of teachers in the data set.

#### Impact of the Situational Factors on Engagement Levels

##### Impact on the Average Number of Days Visiting the App

**Figure 3:**
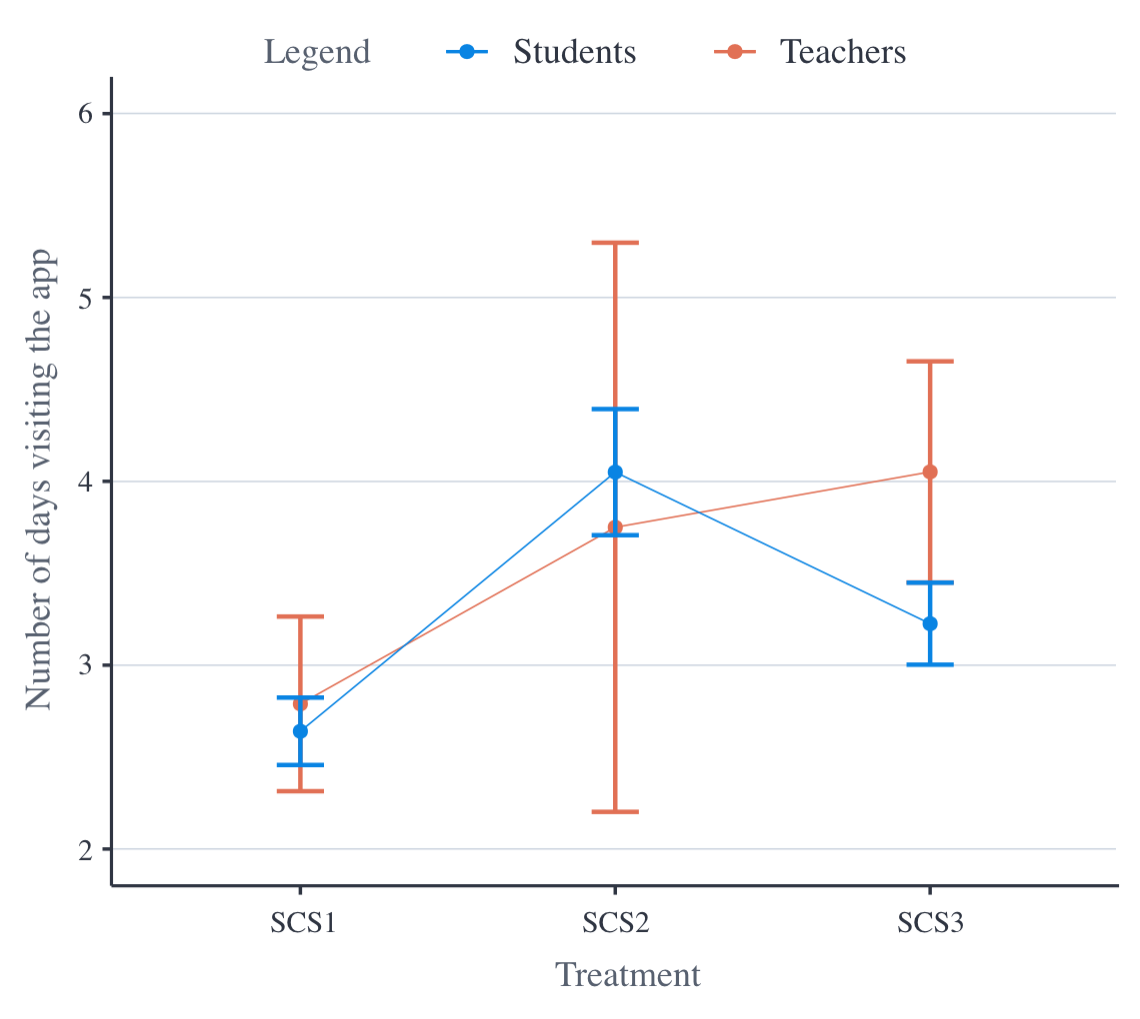
Mean plot of the number of days participants have been visiting the app per treatment. SCS1: first social comparative setting; SCS2: second social comparative setting; SCS3: third social comparative setting.

Figure 3 displays the number of days participants were visiting the app on average, per treatment. Note that the sloped horizontal lines are a visual aid to highlight the differences between the treatment group averages.

From the second set of statistical analyses, it was found that students’ engagement dropped over time (ie, −0.740 days visiting the app per wave; *P*<.001) and also declined during holidays (ie, −0.595 days visiting the app; *P*<.001). In addition, it was found that students in SCS2 were significantly (*P*<.001) visiting the app more often (ie, +0.469 days) than students in SCS1. Further, students in SCS3 were visiting the app more often (ie, +0.215 days) than students in SCS1; however, this difference was not significant. Finally, students in SCS2 were visiting the app more often (ie, +0.255 days) than students in SCS3; however, this difference was not significant. No significant interaction effects were detected, and all treatments were equally impacted by holidays and time.

From the second set of statistical analyses, it was also found that teachers’ engagement also decreased over time (ie, −0.763 days visiting the app per wave; *P*<.001). No additional significant (interaction) effects were observed. Teachers in SCS2 seemed to be visiting the app more often (ie, +1.250 days) than teachers in SCS1, teachers in SCS1 seemed to be visiting the app more often than teachers in SCS3 (ie, +0.173 days), and teachers in SCS3 also seemed to be visiting the app less often (ie, −1.423) than teachers in SCS2; however, none of these differences were reported to be significant.

##### Impact on the Average Number of Unique Activities

**Figure 4:**
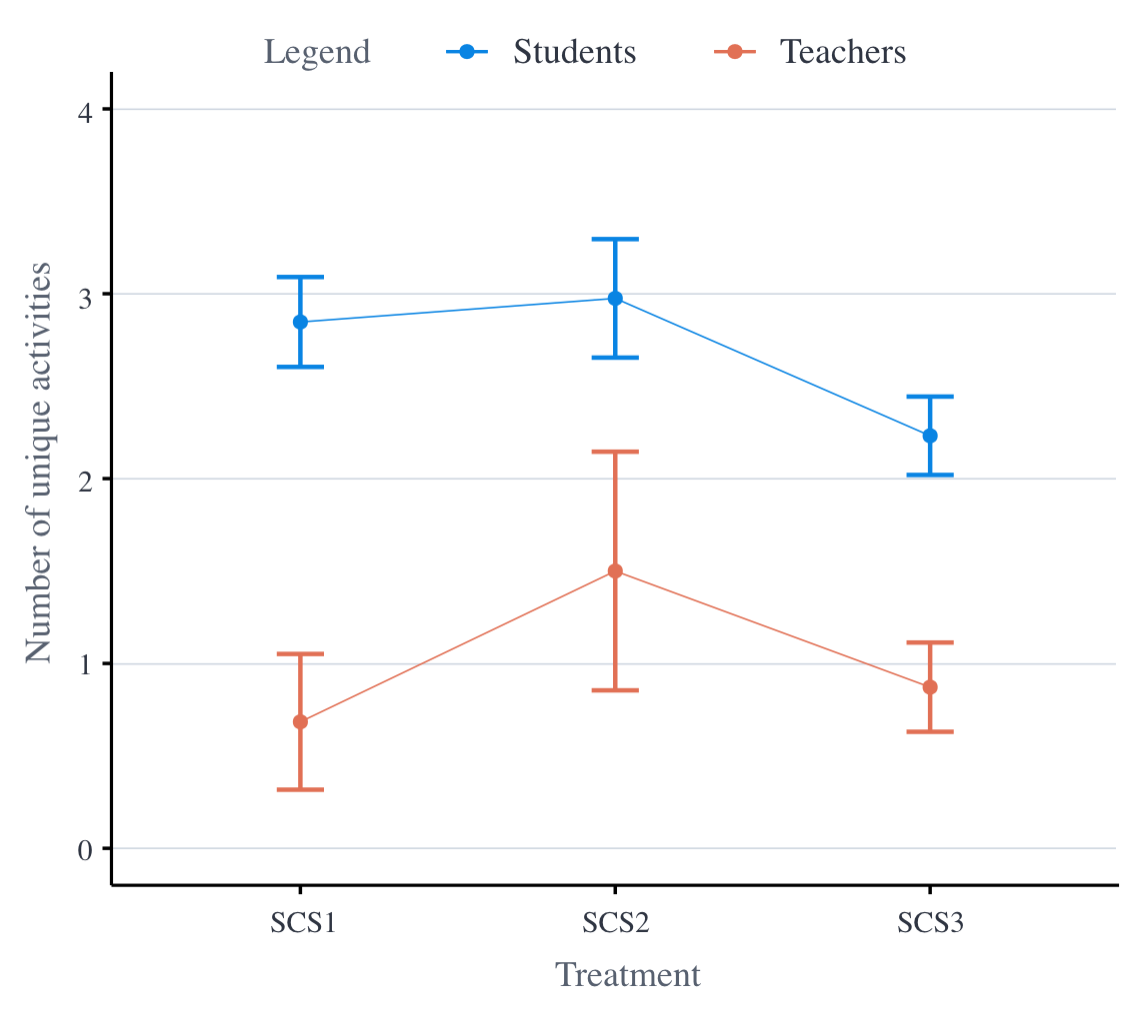
Mean plot of the number of unique activities participants have performed per treatment. SCS1: first social comparative setting; SCS2: second social comparative setting; SCS3: third social comparative setting.

Figure 4 displays the average number of unique activities that participants performed per treatment. Multimedia Appendix 3 displays an overview of the number of times students and teachers performed each prescribed activity.

From the second set of statistical analyses, it was also found that the number of activities students performed decreased over time (ie, −1.249 activities per wave; *P*<.001) and during holidays (ie, −2.611 activities; *P*<.001). In addition, it was found that students in SCS2 performed, on average, more unique activities than students in SCS1 (ie, +0.457 activities); however, this difference was not significant. Students in SCS3, on the other hand, performed fewer unique activities, on average, than students in SCS1 (ie, −0.611 activities); however, this difference was not significant. Students in SCS2 performed significantly more activities, on average, than students in SCS3 (ie, +1.068 activities; *P*=.004). No significant interaction effects were detected; all treatments were equally impacted by holidays and time (ie, wave number).

From the second set of statistical analyses, it was found that the number of activities teachers performed also decreased over time (ie, −0.067 activities per wave; *P*<.001). Teachers, on average, performed fewer unique activities during holidays (ie, −0.019 activities); however, this difference was not significant. In addition, teachers in SCS2 performed more unique activities than teachers in SCS1 (ie, +1.227 activities; *P*=.04); teachers in SCS3 seemed to have performed slightly more unique activities than teachers in SCS1 (ie, +0.035 activities), although this difference was not significant; and teachers in SCS2, on average, performed more unique activities than teachers in SCS3 (ie, +1.192 activities; *P*=.09), although this difference was only close to significance.

#### Impact of the Individual Factors on Student Engagement Levels

##### Impact on the Average Number of Days Visiting the App

To analyze the impact of individual factors on the average number of days participants checked the app, the model selection procedure selected a final model with four predictors. From this final model, it was found that students’ perception of closeness to their teachers had a positive impact on the average number of days they were visiting the app (ie, +1.162 days; *P*=.03). In addition, it was found that the personality traits conscientiousness (+1.444 days; *P*=.02) and openness to experience (+1.398; *P*=.09) had a positive impact on the number of days students checked the app, although the impact of the latter personality trait was not significant. Finally, it was found that the personality trait extraversion had a negative impact on the number of days students were visiting the app (ie, −0.743 days; *P*=.03).

##### Impact on the Average Number of Unique Activities

To analyze the impact of individual factors on the average number of unique activities performed by participants, the model selection procedure selected a final model with three predictors. In particular, it was found that the students’ perception of closeness to their teachers had a positive impact on the number of unique activities they performed (ie, +0.095 activities; *P*=.002). On the other hand, it was also found that the students’ perception of closeness to their peers had a negative impact on the number of unique activities they performed (ie, −0.103 activities; *P*=.002). Finally, it was found that the students’ perception of the relevance of the prescribed activities had a positive impact on the number of unique activities they performed (ie, +0.047 activities; *P*=.05).

## Discussion

### Principal Findings

The aim of this study is to evaluate the implementation of social comparison (ie, either fostering intra- or intergroup comparisons) that is most effective in engaging preadolescent students in a health promotion campaign. Overall, our results indicated that students seem to monitor the intergroup competitions (ie, SCS2 and SCS3) more closely than the intragroup competition (ie, SCS1), as they, on average, checked the mHealth app more often when engaged in team-based comparisons. This result supports our hypothesis that an intergroup competition, with both its collaborative and competitive aspects, can better sustain engagement of students with an mHealth intervention than an intragroup competition, which involves only competitive aspects. In an intergroup competition, active players are more likely to discuss the position of their class on the leaderboard and encourage other class members to engage in the competition, as their own success (ie, winning the competition) depends on the performance of their class members. On the other hand, in an intragroup competition, it would have actually been beneficial for a student to be the only active player, as only the absolute winner would receive a small gift. Therefore, active players in the intragroup competition had no incentive to encourage other players to engage in the competition. Finally, the number of competitors was lower in the intergroup competitions than in the intragroup competition, which may have intensified the competition (ie, according to the N effect [21,22]).

In addition, it was found that students did, on average, complete (significantly) more unique activities in SCS2 (compared with SCS1 and SCS3). However, students in SCS3 completed fewest unique activities on average, whereas we expected that SCS3 would trigger, on average, the highest adoption of healthy routines, as we had introduced an additional comparative element in this setting (ie, by explicitly highlighting the performance of teachers).

In summary, we found that students adopted the most healthy routines in an intergroup competition and the fewest healthy routines in an intergroup competition. As a result, it is difficult to explain the difference between SCS2 and SCS3 based on the number of competitors or incentive structure because these were similar in both cases. However, this unexpected result may be explained by other factors. On the one hand, this result may be explained by the locked variable *educational level*. As we had to select a randomized block approach (ie, based on students’ educational level) to distribute our treatments and given that the majority of our data were collected in the first wave (eg, due to increasing dropout over time), the crossover study design may not have prevented that the impact of a certain treatment is bound to a specific educational level.

On the other hand, the difference between the average number of unique activities that students performed in SCS2 and SCS3 may be explained by examining the teacher’s performance in more detail; a plausible interpretation is that an intergroup competition, in which students cooperate with their mentoring teachers to beat other classes, requires actual involvement of the same mentoring teachers. The discrepancy between SCS2 and SCS3 may then be explained by the fact that teachers completed (significantly) more unique activities, on average, in SCS2 than in SCS3 and SCS1. The fluctuations in the number of activities teachers performed were not controlled for; however, in a social comparative setting where students are likely to draw inspiration from their teacher’s actions [20], these coincidences can have an effect. By coincidence, teachers did not perform many activities in SCS1. Still, in this intragroup competition, students were probably sufficiently motivated by other active students to perform the healthy routines. It so happened that teachers were also passive in setting SCS3; although in this intergroup setting, their behavior probably demotivated students (who depended on them to pull up the class average and inspire other passive students in their class). It so happened that, compared with those in both SCS1 and SCS3, the teachers in SCS2 were actually, on average, performing (significantly) more unique activities. As a result, these teachers could have inspired their students, which explains the higher number of unique activities students performed on average.

Furthermore, when evaluating the individual factors that have influenced students’ engagement levels, it was found that students’ perception of closeness to their teachers had a positive impact on the average number of days they were visiting the app and the average number of unique activities they completed. It is likely that students who *feel closer to* their teachers participate more actively because their teachers have especially invited them to participate. This result supports the claim that teachers potentially serve as positive role models for students [14].

On the other hand, it was also found that students’ perception of closeness to their peers had a negative impact on the number of unique activities they performed. Furthermore, in accordance with the findings of Beach and Tesser [25], it was found that students’ perception of the relevance of the prescribed activities had a positive impact on the average number of unique activities students performed. It was also found that the personality trait conscientiousness had a positive impact on the average number of days students checked the mHealth app. However, in contrast to the findings of Buunk and Gibbons [33], it was found that the personality trait openness to experience actually had a positive impact on the number of days students checked the app. This may be explained by the context in which our study was executed; the health promotion campaign was advertised as a rather alternative form of education and is, therefore, likely perceived by students as *something new*. Finally, it was found that the personality trait extraversion had a negative impact on the number of days students were visiting the app. The negative impact of extraversion on students’ engagement may be explained by the observation that extraverts are more easily bored [36] and, therefore, quit the mHealth intervention earlier than introverts.

Finally, it must be noted that engagement levels with the intervention dropped faster over time than expected. The spring break seemed to have a dramatic impact on students’ engagement levels. In addition, teachers seemed to have been unable to drag their students’ attention back to the health promotion campaign after the holiday period, although their role was implied to be important in raising campaign awareness.

### Limitations

This study did not actively control fluctuations in the engagement levels of teachers (eg, the number of activities they performed). The diverse behavior of teachers has likely influenced the engagement levels of students to some extent. In addition, as the focus of this study was on students’ engagement, teachers did not fill out the posttest survey, which means that no qualitative data were collected on how they perceived the different treatments. Finally, no data were recorded on the number of reviews of the app’s leaderboard teachers had actually hosted during plenary class settings.

Furthermore, although students did fill out the posttest survey, the degree of social relationships between the students was unclear at the start of the experiment. As a result, we could not assess what preadolescents were befriended, what students were most popular, and what teachers were beloved. Potentially, this analysis could have helped to target the most influential subjects and drag their attention back to the health promotion campaign after the spring break. Presumably, the most influential subjects could have also triggered the others to continue active participation.

Another weakness of this study is that social comparison was not studied in complete isolation (eg, some external rewards were provided as well). We kept the additional incentives stable across the treatment groups. Still, it is interesting to evaluate social comparison without any other incentives (eg, without the small gifts that were distributed in this study) to obtain a better estimate of the true impact social comparison has on engagement levels with an mHealth app.

Similarly, this study evaluated the impact of our intervention on a particular target group (ie, preadolescents) within a specific context (ie, the school environment). It is likely that the results will translate to other audiences and contexts because social comparison and its derivatives are universal processes [6-8]; however, it remains unclear what its impact on health behavior would be in different settings.

### Future Work

A follow-up study should control the engagement levels of teachers (eg, by controlling the number of activities they perform) to analyze the exact impact of either passive or active teachers in intragroup and intergroup competitions. Further research is also needed to evaluate whether teachers are sufficiently strong positive role models for preadolescents. It has been demonstrated that social media influencers can serve as alternative role models by, for example, enhancing the dissemination of public health messages [37]. Therefore, a follow-up study could potentially benefit from social media influencer involvement. Finally, future studies may have students create their own teams (eg, in intergroup competitions). It was observed that people interact with different social networks (eg, a network of people for physical interaction and a network of people for sharing web-based messages) [38]. It would be interesting to investigate what type of network (ie, social comparative setting) is most effective in promoting the adoption of health routines among preadolescents.

Finally, we encourage studies evaluating persuasive strategies other than social comparison in this target population, such that we can compare the impact of individual behavior change techniques on preadolescents. We suggest that scholars should also conduct these studies within a relatively safe environment for preadolescents, such as their high schools. Although mHealth tools are deployed to promote something good in its users (ie, a person’s health), the persuasive nature of behavior change techniques may potentially threaten an individual’s freedom of conduct. Ethical guidelines for developing moral mHealth tools for preadolescents are still in their infancy. Dedicated research within the ethics community is trying to answer questions on the moral aspects of the development of mHealth tools [39], and guidelines for developing moral artificial intelligence interventions are emerging [40], which may also apply to specific mHealth interventions. We welcome additional ethical guidelines for the development of mHealth tools and the execution of empirical studies to evaluate these tools.

### Conclusions

When using social comparison to engage preadolescents in a health promotion campaign using an mHealth tool, an intergroup competition—rather than an intragroup competition—can increase preadolescents’ passive engagement with mHealth apps. However, an intergroup competition, as compared with an intragroup competition, does not necessarily result in preadolescents performing more unique activities on average. The active involvement of a role model (eg, a teacher) can influence the average number of unique activities preadolescents perform in an intergroup setting. For example, if the role model is active, preadolescents seems more likely to actively participate as well, because preadolescents are likely to draw inspiration from the actions of their role models. Moreover, preadolescents’ perception of closeness to their role model seems to amplify this effect.

From this study, it is concluded that HPS interventions can use social dynamics to engage preadolescent students in healthier routines. However, additional behavior change strategies seem necessary to sustain students’ engagement over time. In this process, an especially important role seems reserved for the teachers who serve as role models for their students and can potentially inspire them if they are actively involved in the HPS intervention themselves as well.

## Appendix

Multimedia Appendix 1 Posttest survey.

Multimedia Appendix 2 An overview of prescribed activities per wave.

Multimedia Appendix 3 An overview of the number of times different prescribed activities were performed.

## Abbreviations

HPS: Health Promoting School
mHealth: mobile health
SCS1: first social comparative setting
SCS2: second social comparative setting
SCS3: third social comparative setting
VMBO: Voorbereidend middelbaar beroepsonderwijs
