# Supplementary material for: Preadolescent Students’ Engagement With an mHealth Intervention Fostering Social Comparison for Health Behavior Change: Crossover Experimental Study
Source: J Med Internet Res. 2021 Jul 29;23(7):e21202. doi: 10.2196/21202 (PMC8367116; doi:10.2196/21202)
Supplement: Multimedia Appendix 1 [file jmir_v23i7e21202_app1.pdf]

GAMEBUS

* 1. Wat is jouw e-mailadres (bij voorkeur jouw @udenscollege.nl adres)?

* 2. Welke afbeelding beschrijft het beste de band die jij met je ***mentor/coach*** hebt?

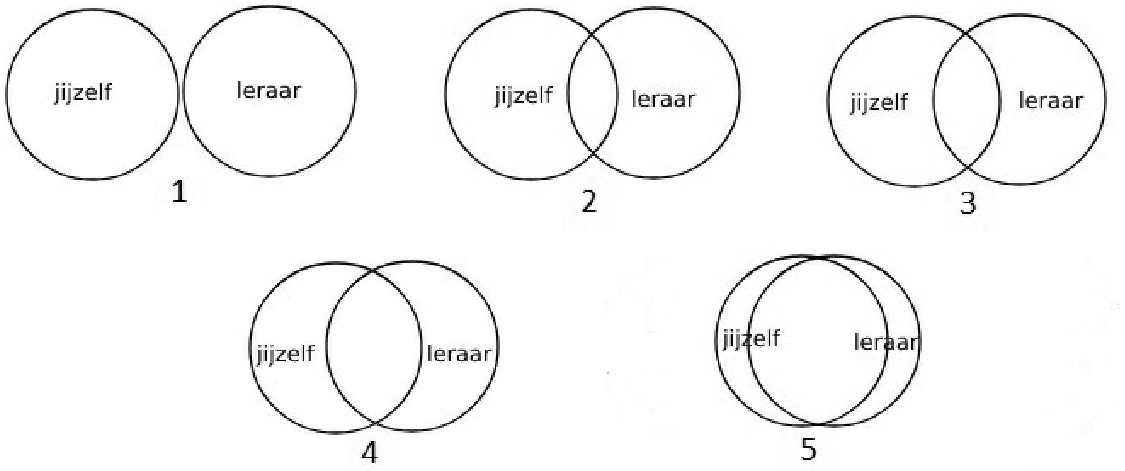

- 1
- 2
- 3
- 4
- 5

* 3. Welke afbeelding beschrijft het beste de band die jij met je ***medeleerlingen*** hebt?

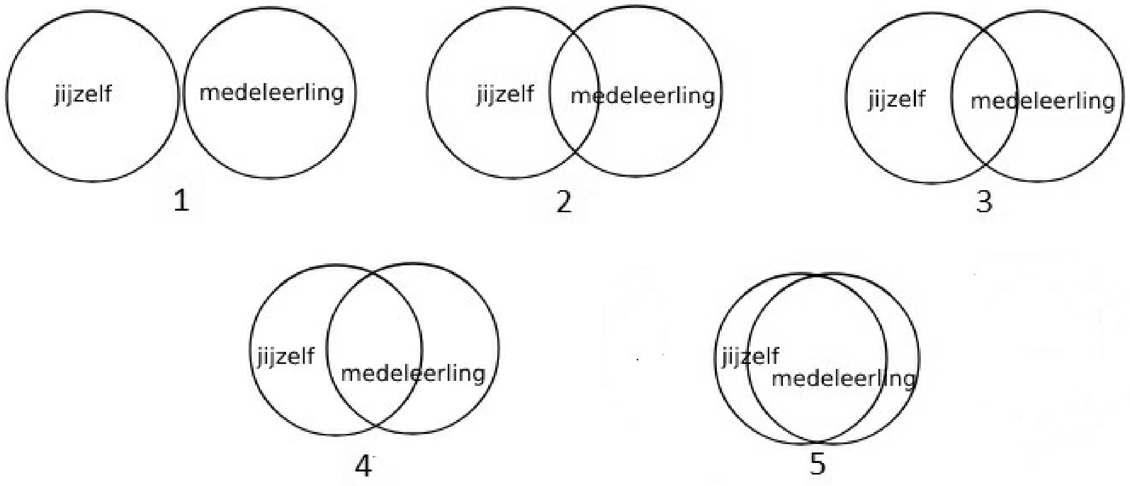

- 1
- 2
- 3
- 4
- 5

* 4. Welke afbeelding beschrijft het beste de band die jij over het algemeen met ***anderen*** hebt?

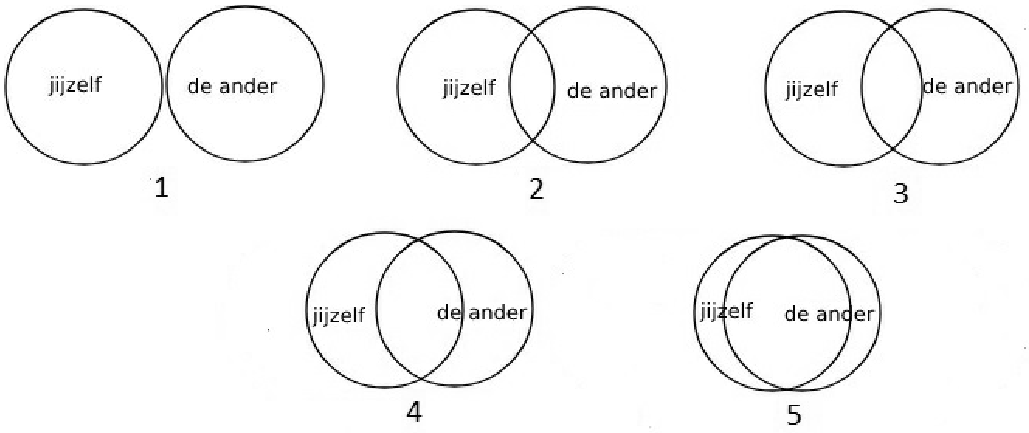

- 1
- 2
- 3
- 4
- 5

* 5. Als ik mijzelf vergelijk met *mijn mentor/coach* op het gebied van sport vind ik mijzelf

Slechter Beter

* 6. Als ik mijzelf vergelijk met *mijn mentor/coach* vind ik mijzelf

Anders — Hetzelfde

* 7. Als ik mijzelf vergelijk met *medeleerlingen* op het gebied van sport vind ik mijzelf

Slechter — Beter

8. Als ik mijzelf vergelijk met *medeleerlingen* vind ik mijzelf

Anders — Hetzelfde

* 9. Als ik mijzelf vergelijk met anderen op het gebied van sport vind ik mijzelf

Slechter — Beter

* 10. Als ik mijzelf vergelijk met *anderen* vind ik mijzelf

Anders — Hetzelfde

* 11. Ik word geïnspireerd door de volgende personen

	Helemaal eens	Een oneens	Neutraal	Oneens	Helemaal oneens
Je mentor/coach	◯	◯	◯	◯	◯
Medeleerlingen	◯	◯	◯	◯	◯
Iemand die jou inspireert. Denk aan een vlogger of voetballer	◯	◯	◯	◯	◯

* 12. Word jij geïnspireerd door iemand anders?

◯ Nee

◯ Ja, namelijk:

* 13. Van wie zou jij het liefste willen winnen?

◯ Leraren

◯ Andere klassen

* 14. Van wie zou jij het liefste ***niet*** willen verliezen?

- Leraren
- Andere klassen

* 15. Ik ben het middelpunt van een feestje

- Helemaal oneens
- Oneens
- Neutraal
- Eens
- Helemaal mee eens

* 16. Ik leef mee met de gevoelens van anderen

- Helemaal oneens
- Oneens
- Neutraal
- Eens
- Helemaal mee eens

* 17. Ik doe klusjes meteen

- Helemaal oneens
- Oneens
- Neutraal
- Eens
- Helemaal mee eens

* 18. Ik heb regelmatig stemmingswisselingen

Stemmingswisselingen: Een wisseling in je stemming of bui. Bijvoorbeeld dat je van heel verdrietig naar heel blij gaat in een korte tijd.

- Helemaal oneens
- Oneens
- Neutraal
- Eens
- Helemaal mee eens

* 19. Ik heb een levendige fantasie

- Helemaal oneens
- Oneens
- Neutraal
- Eens
- Helemaal mee eens

* 20. Ik praat niet veel

- Helemaal oneens
- Oneens
- Neutraal
- Eens
- Helemaal mee eens

* 21. Ik ben niet geïnteresseerd in de problemen van anderen

- Helemaal oneens
- Oneens
- Neutraal
- Eens
- Helemaal mee eens

* 22. Ik vergeet vaak dingen op hun goede plek terug te leggen

- Helemaal oneens
- Oneens
- Neutraal
- Eens
- Helemaal mee eens

* 23. Ik ben meestal ontspannen

- Helemaal oneens
- Oneens
- Neutraal
- Eens
- Helemaal mee eens

* 24. Ik ben niet geïnteresseerd in abstracte ideeën.
Abstract: Een abstract begrip is iets dat je niet kunt zien of aanraken. Een voorbeeld van een abstract begrip is liefde of klimaat. Wiskunde is een abstract vak.

- Helemaal oneens
- Oneens
- Neutraal
- Eens
- Helemaal mee eens

* 25. Ik praat op feestjes met veel verschillende mensen

- Helemaal oneens
- Oneens
- Neutraal
- Eens
- Helemaal mee eens

* 26. Ik voel andermans emoties aan

- Helemaal oneens
- Oneens
- Neutraal
- Eens
- Helemaal mee eens

* 27. Ik ben gesteld op orde

- Helemaal oneens
- Oneens
- Neutraal
- Eens
- Helemaal mee eens

* 28. Ik ben snel overstuur

- Helemaal oneens
- Oneens
- Neutraal
- Eens
- Helemaal mee eens

* 29. Ik heb moeite om abstracte ideeën te begrijpen

Abstract: Een abstract begrip is iets dat je niet kunt zien of aanraken. Een voorbeeld van een abstract begrip is liefde of klimaat. Wiskunde is een abstract vak.

- Helemaal oneens
- Oneens
- Neutraal
- Eens
- Helemaal mee eens

* 30. Ik houd me op de achtergrond

Achtergrond: Als je niet graag op de voorgrond staat dan blijf je liever op de achtergrond. Bijvoorbeeld wanneer je tijdens de schoolmusical liever een bijrol hebt dan de hoofdrol

- Helemaal oneens
- Oneens
- Neutraal
- Eens
- Helemaal mee eens

* 31. Ik ben niet echt geïnteresseerd in anderen

- Helemaal oneens
- Oneens
- Neutraal
- Eens
- Helemaal mee eens

* 32. Ik maak een puinhoop van dingen

- Helemaal oneens
- Oneens
- Neutraal
- Eens
- Helemaal mee eens

* 33. Ik voel me zelden droevig

- Helemaal oneens
- Oneens
- Neutraal
- Eens
- Helemaal mee eens

* 34. Ik heb weinig fantasie

- Helemaal oneens
- Oneens
- Neutraal
- Eens
- Helemaal mee eens

GAMEBUS

Tevredenheid met de campagne & het gebruik van GameBus

* 35. Ik vond de activiteiten waar ik punten voor kon krijgen door GameBus leuk

- Helemaal niet mee eens
- Niet mee eens
- Neutraal
- Mee eens
- Helemaal mee eens

* 36. Ik was in staat om de GameBus challenges die punten opleverde uit te voeren

- Helemaal niet mee eens
- Niet mee eens
- Neutraal
- Mee eens
- Helemaal mee eens

* 37. Het aantal punten dat gegeven werd voor de verschillende GameBus challenges was eerlijk verdeeld

- Helemaal niet mee eens
- Niet mee eens
- Neutraal
- Mee eens
- Helemaal mee eens

* 38. Ik had het gevoel dat mijn medeleerlingen niet vals speelden

- Helemaal niet mee eens
- Niet mee eens
- Neutraal
- Mee eens
- Helemaal mee eens

* 39. Hoe waarschijnlijk is het dat je de GameBus-app zult aanbevelen aan een vriend of familielid?

Zeer onwaarschijnlijk — Zeer waarschijnlijk

* 40. Welke ervaring met de GameBus campagne wilt u delen?
