# Supplementary material for: Preadolescent Students’ Engagement With an mHealth Intervention Fostering Social Comparison for Health Behavior Change: Crossover Experimental Study
Source: J Med Internet Res. 2021 Jul 29;23(7):e21202. doi: 10.2196/21202 (PMC8367116; doi:10.2196/21202)
Supplement: Multimedia Appendix 2 [file jmir_v23i7e21202_app2.pdf]

Activities							Waves					
ID	Theme	Activity name	Points	Frequency			1	2	3	4	5	6
PA01	Physical activity	Wrestle arms with someone of at least 40+	3	5	× per	day	1					
PA02	Physical activity	Play tag with Picoo	5	1	× per	wave	1					
PA03	Physical activity	Mimic this yoga posture: □	2	1	× per	day	1					
PA04	Physical activity	Selfie of you and your friends sweating	3	1	× per	day	1		1		1	
PA05	Physical activity	Visit a sport's facility in Uden	2	5	× per	day	1			1		
PA06	Physical activity	Create a human pyramid, as high as possible	6	5	× per	day	1				1	
PA07	Physical activity	Go rolling (hint: rollerblading, rollerblading, etc.)	2	1	× per	day		1				
PA08	Physical activity	Photograph the start of a mountain bike trial at De Mastworp	3	1	× per	wave		1				
PA09	Physical activity	Play a game of dogeball	4	2	× per	week			1			
PA10	Physical activity	Show your most advanced moves on a punching bag	3	1	× per	day				1		
PA11	Physical activity	Write down the names of at least 10 different apps that encourage people to keep moving	4	1	× per	wave				1		
PA12	Physical activity	Visit a training in self-defense at MC Uden	5	1	× per	week				1		
PA13	Physical activity	Play soccer with at least three of your friends	3	1	× per	day					1	
PA14	Physical activity	Play a game of 'conquer the flag'	4	2	× per	week					1	
PA15	Physical activity	Do 25 sit-ups	2	1	× per	day						1
PA16	Physical activity	Perform the trick 'around the world' with a ball	2	1	× per	day						1
PA17	Physical activity	Complete an interval training from Youtube	4	1	× per	day						1
PA18	Physical activity	Do as many Fortnite dances as possible within 1 minute	4	1	× per	day						1
NU01	Nutrition	Join the healthy smoothie workshop	5	1	× per	wave	1					
NU02	Nutrition	Peel an (unbroken) apple peel of at least 20 centimeters	2	2	× per	day	1					
NU03	Nutrition	Photograph yourself with a healthy meal	3	1	× per	day		1				
NU04	Nutrition	Make the tastiest salad	3	1	× per	day		1				
NU05	Nutrition	Photograph a shopping cart with ingredients for a healthy meal	4	1	× per	day		1				
NU06	Nutrition	Eat a piece of fruit	2	1	× per	day		1		1		1
NU07	Nutrition	Cook a healthy meal for your parents / guardians	5	1	× per	day			1			
NU08	Nutrition	Make the most delicious healthy sandwich	3	1	× per	day				1		
NU09	Nutrition	Visit a greengrocer in Uden	3	1	× per	day				1		
NU10	Nutrition	Research how many calories you can have in a day	3	1	× per	wave						1
NU11	Nutrition	Make the healthiest menu	4	1	× per	week						1
NU12	Nutrition	Take a selfie at the strawberry drive-in	4	1	× per	day						1
NU13	Friends, love, and	Take a group photo with your entire class	4	1	× per	week	1					
NU14	Friends, love, and	Take the bike to Bedaf (i.e., a park) with friends	2	1	× per	day			1			
FL01	Friends, love, and	Photograph yourself with someone who is at least 10 years older / younger than you	1	3	× per	day			1			
FL02	Friends, love, and	Together with friends, form a word of at least seven letters with your bodies	5	3	× per	day			1			
FL03	Friends, love, and	Dress up as crazy as possible with friends	4	1	× per	day			1			
FL04	Friends, love, and	Play a game of pool in MC Uden	4	2	× per	week			1			
FL05	Friends, love, and	Photograph yourself with a couple who have been married for more than 20 years	4	2	× per	day			1			
FL06	Friends, love, and	Take a group photo on the mosaic bench	2	1	× per	day					1	
FL07	Friends, love, and	Go to a care home and help an elderly person do the groceries	5	1	× per	day					1	
FL08	Friends, love, and	Make 5 random people laugh	4	3	× per	day					1	
FL09	Friends, love, and	Take a photo with your (best) friend (maximum 2 people in the photo)	1	3	× per	day					1	
FL10	Friends, love, and	Work out with someone who is more than 50 years older than you in De Beweegtuin	5	1	× per	day					1	
FL11	Friends, love, and	Via wordzoekers.org, make a word search puzzle about 'falling in love'	4	1	× per	week						1
SD01	Stress, drugs, alcol	Visit the workshop on stress and watch the documentary 'Stress To Impress'	5	1	× per	wave	1					
SD02	Stress, drugs, alcol	Visit the workshop on alcohol by the Public Health Service, and make a selfie with the alcohol goggles	5	1	× per	wave	1					
SD03	Stress, drugs, alcol	Visit the workshop on smoking by the Public Health Services	5	1	× per	wave	1					
SD04	Stress, drugs, alcol	Find a reliable article about abuse on social media (for example, on cyberbullying)	4	1	× per	week		1				
SD05	Stress, drugs, alcol	Play a game of FIFA on the Playstation in the MC Uden	4	1	× per	week		1				
SD06	Stress, drugs, alcol	Create a (really working) smoke robot	6	1	× per	wave		1				
SD07	Stress, drugs, alcol	Make a drawing, collage or portrait of the consequences of (too much) Stress, drugs, alcohol and Stress, drugs, alcohol and gaming	6	1	× per	week				1		
SD08	Stress, drugs, alcol	Via wordzoekers.org, make a word search puzzle about 'stimulants & drugs'	4	1	× per	week				1		
NU15	Nutrition	Eet een biologisch snoepje van snoepwinkel 'Veel Liefs': bijvoorbeeld zouthout	5	1	× per	week						
SD09	Stress, drugs, alcol	Lever je telefoon voor minimaal vier uur in bij de conciërge	6	1	× per	day						
SD10	Stress, drugs, alcol	Voer de stresstest uit op: en upload een screenshot van jouw score	2	1	× per	day						
