# Supplementary figures and images for: Preadolescent Students’ Engagement With an mHealth Intervention Fostering Social Comparison for Health Behavior Change: Crossover Experimental Study
Source: J Med Internet Res. 2021 Jul 29;23(7):e21202. doi: 10.2196/21202 (PMC8367116; doi:10.2196/21202)

Number of times students and teachers have performed each unique activity

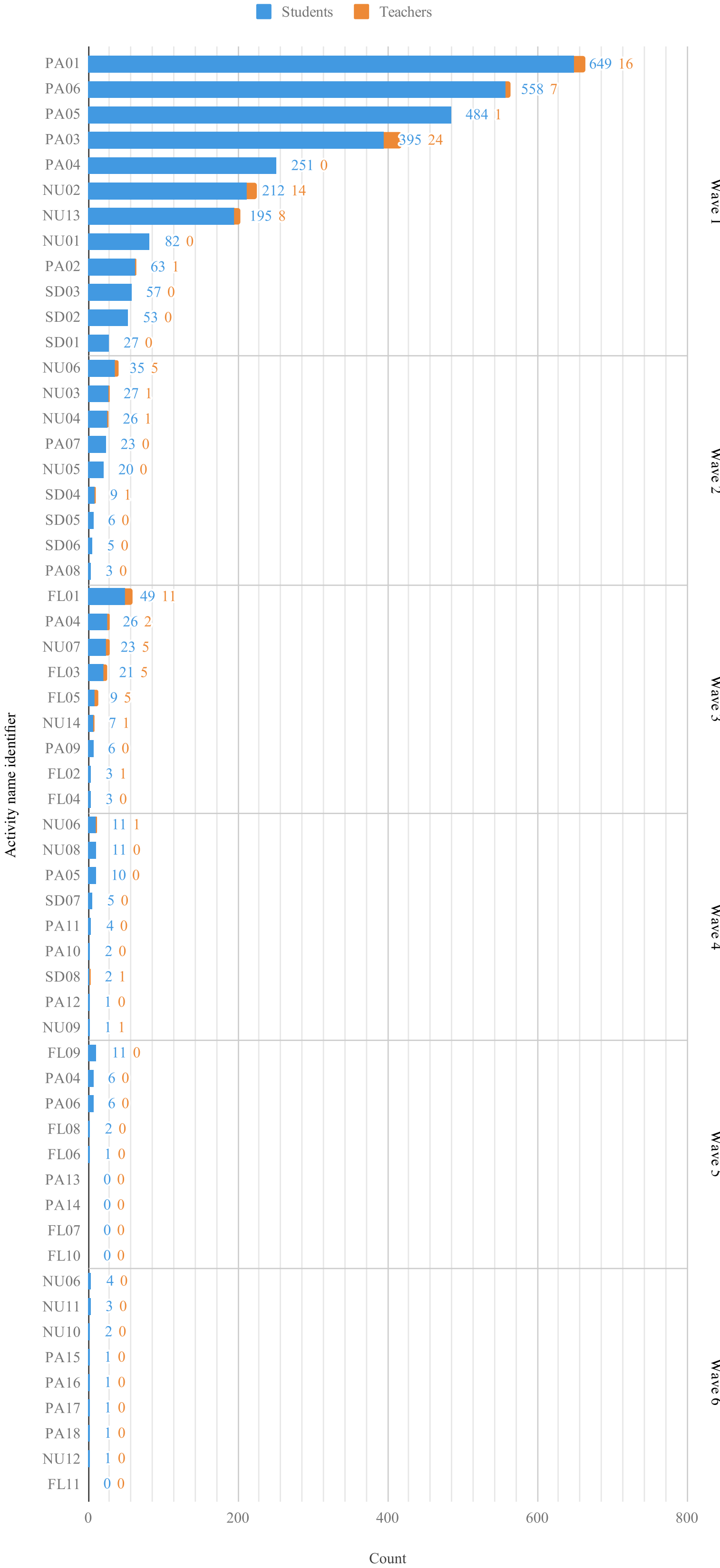

Supplement: Multimedia Appendix 3 [file jmir_v23i7e21202_app3.pdf]
